# Vitamin D and Inflammatory Bowel Disease

**DOI:** 10.1155/2015/470805

**Published:** 2015-04-27

**Authors:** Marco Ardesia, Guido Ferlazzo, Walter Fries

**Affiliations:** ^1^Internal Medicine, Department of Clinical and Experimental Medicine, University of Messina, Messina, Italy; ^2^Laboratory of Immunology and Biotherapy, Department of Human Pathology, University of Messina, Messina, Italy; ^3^Clinical Unit for Chronic Bowel Disorders, IBD-UNIT, Department of Clinical and Experimental Medicine, University of Messina, Via Consolare Valeria 1, 98125 Messina, Italy

## Abstract

Vitamin D deficiency has been recognized as an environmental risk factor for Crohn's disease since the early 80s. Initially, this finding was correlated with metabolic bone disease. Low serum 25-hydroxyvitamin D levels have been repeatedly reported in inflammatory bowel diseases together with a relationship between vitamin D status and disease activity. Subsequently, low serum vitamin D levels have been reported in various immune-related diseases pointing to an immunoregulatory role. Indeed, vitamin D and its receptor (VDR) are known to interact with different players of the immune homeostasis by controlling cell proliferation, antigen receptor signalling, and intestinal barrier function. Moreover, 1,25-dihydroxyvitamin D is implicated in NOD2-mediated expression of defensin-*β*2, the latter known to play a crucial role in the pathogenesis of Crohn's disease (IBD1 gene), and several genetic variants of the vitamin D receptor have been identified as Crohn's disease candidate susceptibility genes. From animal models we have learned that deletion of the VDR gene was associated with a more severe disease. There is a growing body of evidence concerning the therapeutic role of vitamin D/synthetic vitamin D receptor agonists in clinical and experimental models of inflammatory bowel disease far beyond the role of calcium homeostasis and bone metabolism.

## 1. Introduction

Vitamin D is a fat-soluble vitamin whose active form, calcitriol or 1,25-dihydroxyvitamin D_3_ (1,25(OH)_2_D_3_), regulates bone, calcium, and phosphorus metabolism [1]. However, vitamin D also influences immune system function, and deficiency has been recognized as an environmental risk factor for autoimmune diseases like Crohn's disease (CD) [2].

In humans, vitamin D may be obtained from two sources: diet (as fat-soluble vitamin) and by ultraviolet- (UV-) mediated synthesis in the epidermal layer of the skin where UV-rays promote photolytic cleavage of 7-dihydrocholesterol (7-HDC) into vitamin D_3_ [3]. The latter is the most important source of this metabolite and, at this point, vitamin D can be considered as a hormone [4]. After production, vitamin D is activated by a two-step hydroxylation, first in the carbon 5-position by 25-hydroxylase in the liver then by 1*α*-hydroxylase in the kidney: this active metabolite exerts its functions by interacting with the vitamin D receptor (VDR), a receptor that belongs to the superfamily of nuclear hormone receptors [1]. Binding to VDR leads to the transcription of several vitamin D-response genes, located on single loci [5]. Various tissues and, especially, immune-related cells express VDRs and are able to produce 1,25(OH)_2_D_3_. This implies that the vitamin exerts its action beyond its classic hormonal-endocrine function tending towards an autocrine role [6].

## 2. Vitamin D and Its Role in Immune Regulation

Vitamin D affects the immune system acting at various levels, such as antibacterial response, antigen presentation, and regulation of adaptive and innate immunity. Genome-wide analysis has revealed that a large number of genes are influenced by vitamin D levels [7]. VDRs have been discovered in almost all immune cells as activated or naïve CD4^+^ and CD8^+^ T cells, B cells, neutrophils, and antigen-presenting cells (APCs) such as dendritic cells and macrophages. In particular, vitamin D_3_ enhances the chemotactic and phagocytic responses of macrophages and production of antimicrobial proteins, such as cathelicidin, inhibits the surface expression of the MHC-II-complex antigen and costimulatory molecules and downregulates the production of many proinflammatory cytokines, such as interleukin- (IL-) 1, IL-6, IL-8, and TNF-*α* [4, 8]. An experimental study demonstrated that transferring CD8^+^ T cells isolated from the spleen of wild type (WT) and IL-10 KO mice into immunodeficient Rag KO recipients, that is, mice with no mature B or T cells, did not induce colitis, whereas transferring CD8^+^ T cells from VDR KO mice led to colonic inflammation, and transferring CD8^+^ T cells from IL-10/VDR KO mice led to fulminant colitis. These data indicate that expression of VDR is required to prevent replication of quiescent CD8^+^ T cells and that the lack of VDR induced the formation of more aggressive T cells [9]. Another study evaluated the difference of protein expression in the small intestinal mucosa between WT mice and VDR KO mice identifying a higher expression of proteins involved in cell adhesion, proliferation, and migration and stress response in VDR KO mice. The authors conclude that vitamin D and VDR play a direct, or indirect role, in balancing these functions [10].

Vitamin D/VDR status regulates development, function, and balance of T-lymphocytes dampening T-helper- (Th-) 1 cell function and cytokine patterns (IL-2 and interferon-*γ* (IFN-*γ*)) by enhancing the Th-2 cell response (IL-4, IL-5, and IL-10) [11]; moreover 1,25(OH)_2_D_3_ promotes a regulatory outcome through the inhibition of Th-17 cells and their related cytokines, and the induction of regulatory T cells (Treg) that are protective against autoimmunity, stimulating the expression of the cytotoxic T-associated protein 4 (CTLA-4) and forkhead box P3 (Foxp-3), together with the induction of IL-10 [12, 13]. In addition, 1,25(OH)_2_D_3_ appears to have a chemopreventive role through an antiproliferative action, for example, through VDR-mediated inhibition of the Wnt/beta-catenin pathway [8, 14, 15], inhibiting growth without inducing apoptosis and inducing differentiation in colon cancer cell lines [16, 17].

**Figure 1:**
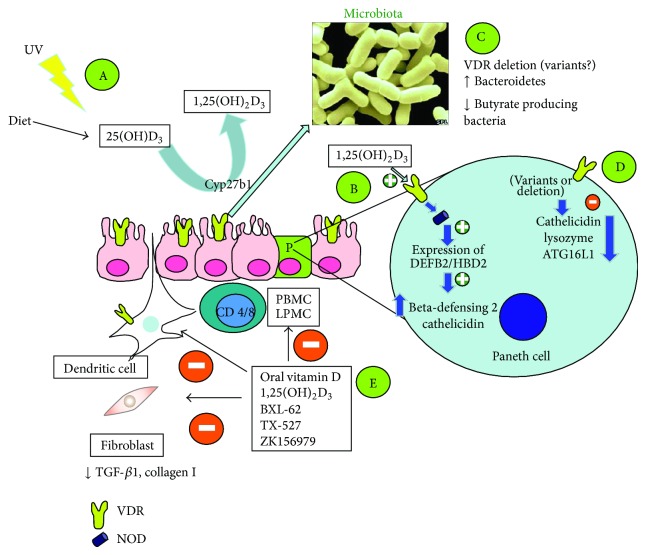
Potential involvement of vitamin D in the pathogenesis of inflammatory bowel disease and immunologic effects of vitamin-D-related therapeutic approaches. Scenario A: reduced UV exposure as risk factor for CD and for hospitalizations and surgery [86]; Scenario B: NOD2 gene transcription is stimulated by 1,25(OH)_2_D_3_/VDR and signaling through NOD2 induces expression of DEFB2/HBD2 which stands for beta-defensin 2 and cathelicidin [20]; Scenarios C and D: variants or loss of function of VDR may lead to changes of the microbiota and reduce host defense by reducing production of cathelicidin, lysozyme, and ATG16L1 protein (autophagy) [21, 22]; Scenario E: experimental studies with vitamin D or its analogues showing inhibitory effects on PBMC, LPMC, dendritic cells, and fibroblasts in terms of cytokine production and differentiation (Table 3). VDR: vitamin D receptor; NOD: nucleotide-binding oligomerization domain.

The molecular and genetic link between CD and the vitamin D/immune system axis may be in part explained by the NOD2 gene (Figure 1). The precise etiology of the inflammatory bowel disease CD is unknown. Like many chronic diseases, there are environmental factors that act on a polygenic background. Variants of the NOD2/CARD15 gene are associated with the development and phenotypic patterns of CD. This gene encodes for a protein of the family of intracellular pattern recognition receptors for bacterial components that play an important role in the innate immune system [18, 19]. Transcription of the NOD2 gene is stimulated by 1,25(OH)_2_D_3_/VDR and signaling through NOD2 induces expression of DEFB2/HBD2 which stands for the antimicrobial peptide beta-defensin 2, and of CAMP which codifies for cathelicidin [20]. In a study on a VDR KO model, a downregulation of the ATG16L1 gene, together with a reduced expression of lysozyme by Paneth cells was reported [21]. These mice had an increased susceptibility to dextran sulfate sodium (DSS) colitis, whereas in human colon samples of low VDR expression correlate with ATG16L1 and a reduction of* Bacteroides* species. This finding implies that alterations of the vitamin D status might interfere with autophagy and alter the antimicrobial barrier of the intestinal mucosa and, consequently, the control of the microbiota [22].

## 3. VDR Polymorphisms in IBD

From the above, it appears that variants of VDR interfere with the immune system and, thus, may contribute to susceptibility to inflammatory bowel disease (IBD) [23, 24]. In fact, VDR polymorphisms have been identified in various diseases, such as cancer [25] or cancer risk [26], asthma [27], and kidney diseases [28]. The best-studied polymorphisms include BsmI (rs1544410), FokI (rs2228570), TaqI (rs731236), and ApaI (rs7975232). However, the results of these still few studies in IBD patients are contradictory (Table 1): for example, no statistical significance compared to controls was found in two studies on IBD patients for BsmI, FokI, TaqI, and ApaI [29, 30] with a borderline significance for heterozygous carriage of the FokI allele [29]. In three Chinese studies on ulcerative colitis (UC) patients [31, 32] and on CD patients, no difference [32] or an association of the Bb genotype of the BsmI variant with UC [31] was reported; whereas no association was found for ApaI, TaqI, and BsmI with CD [33].

In another study on European Caucasian patients, a significantly higher frequency of the TaqI polymorphism (genotype “tt”) was reported in CD compared to UC or HC [23]. This finding was replicated in German IBD patients where the “tt” genotype was significantly more frequent in fistulizing and stenosing CD [24]. Subsequently, always in Caucasians, the finding of a lack of association of ApaI but a more frequent presence of TaqI in male IBD patients was reported [34] and confirmed 3 years later [35].

Concerning BsmI polymorphisms, the BB genotype was more frequent in Ashkenazi UC patients compared to Ashkenazi controls [36]. Finally, in a mixed IBD population investigating all 4 VDR variants, only the Fok I variant (“ff” genotype) was significantly more frequent in IBD patients [37].

Two recent meta-analyses including the same 9 studies with slightly different patient numbers (Table 1) yielded different results [38, 39]; Xue et al. [38] found that the “ff” genotype of FokI was associated with a significant risk for UC in Asians, whereas the “tt” genotype of TaqI was associated with an increased risk for CD in Europeans, but with an increased risk for both diseases, CD and UC, in Asian males. Carriage of the “a” allele (ApaI) resulted protective from CD. In contrast, Wang et al. [39] concluded that there was no association between ApaI, BsmI, and FokI and IBD, whereas subgroup analysis evidenced an increased risk for CD for ApaI and limited to East Asians, for BsmI. Conversely, TaqI variants reduced the risk for UC in Caucasians.

One study examined the influence of VDR polymorphisms on serum vitamin D levels [40] (not included in Table 1) showing a significant association of variants of the TaqI and the signal peptide, CUB domain, and EGF-like 3 (SCUBE3, rs732594) genes, the latter encodes for a protein involved in the VDR pathway, in CD patients, whereas ApaI and SCUBE3 and two variants of PHD finger protein-11 (PHF-11) gene, namely, rs2980 and rs2981, showed a significant association with serum vitamin D levels in CD patients. PHF-11 variants have been shown to be involved with vitamin D levels in other pathologies, such as asthma [41].

Besides investigations on VDR variants, 2 SNPs of the vitamin D-binding protein (DBP), that is, the 416 variant Glu (rs7041) and the 420 variant Lys (rs4588), were analysed. A significantly reduced frequency of the 420 variant Lys was found in IBD patients compared to controls [42].

In conclusion, the influence of VDR variants on IBD risk is still poorly defined. Interesting approaches are represented by investigations on the association between polymorphisms and vitamin D levels and those examining proteins involved in vitamin D-related pathways, but all need further studies and confirmation.

## 4. Vitamin D Status and Related Risk Factors in IBD 

Starting in the late seventies, investigations on the vitamin D status of IBD patients have been carried out with different methodological approaches and results. By comparing IBD patients (CD alone or mixed populations) versus healthy controls (HC), no differences were found for circulating 25(OH)D_3_ concentrations in 6 studies on adult IBD populations [43–48] and in 1 study on a pediatric cohort [49], whereas lower plasma levels were reported in undernourished CD patients [50], in CD patients after intestinal resections [51], in 2 studies on adult, and in 1 study on pediatric CD patients [52–54] and in 3 mixed IBD populations [55–57].

Comparing 25(OH)D_3_ levels between CD and UC patients, no differences were found in 8 studies on adult or pediatric patients in basal conditions [47, 54, 55, 57–61] and in 1 pediatric study on partially vitamin D supplemented patients [62]. Lower levels in CD compared with UC were found in 5 studies [46, 63–66].

Finally, investigations concerning the active form of vitamin D, 1,25(OH)_2_D_3_, reported normal levels after bowel resections in CD [67] but no differences between well- and undernourished CD patients compared to HC or in well-nourished UC patients [50]. Similar findings were reported in a pediatric study including CD, UC and HC [61]. Lower 1,25(OH)_2_D_3_ concentrations compared to controls were found in 2 studies including CD and UC patients [45, 68]. Conversely, elevated levels of 1,25(OH)_2_D_3_ were reported after ileal resections in CD [51]. In this latter study, a positive correlation with 25(OH)D_3_ levels and PTH was reported.

Changing methodology and introducing vitamin D reference values as parameter, the importance of vitamin D in IBD has become more convincing. Defining vitamin sufficiency as serum values above 30 ng/mL, vitamin D insufficiency as values between 10/20 and 30 ng/mL, and vitamin deficiency as concentrations below 10 to 15 ng/mL, data from 27 studies from all over the world were available [44, 46, 52, 53, 56–58, 60, 61, 63, 64, 69–77], 6 of them on cohorts over 100 participants [59, 62, 66, 78–80], and one with more than 1,000 patients [81]. In synthesis, vitamin deficiency was found in 8-100% of patients with CD and in 15-60% of patients with UC, vitamin insufficiency in 12-72.3% in CD or in mixed IBD populations and in 7-64% of UC patients. Five papers [53, 61, 74, 76, 82] differentiated vitamin D levels according to seasonal variations in CD patients reporting vitamin deficiency in 50–76% in winter and in 10–19% in summer months; vitamin insufficiency, where reported [76, 82], was indicated in 73–100% in winter and 55–59% in summer months.

Studies evaluating vitamin D levels in IBD patients were all conducted after disease onset and established diagnosis, but it is not clear if vitamin D deficiency is the cause or a consequence. Pathogenesis of vitamin D hypovitaminosis in patients with IBD may depend on various mechanisms such as decreased exposure to sunlight or oral vitamin D intake, ileal resections leading to malabsorption or a disturbed enterohepatic circulation, and/or increased losses through the gastrointestinal system by protein-losing enteropathy [59].

To identify the reasons for the differences of the vitamin D status, the ability to absorb vitamin D_2_ was evaluated in a study by Farraye et al. [77] comparing CD patients and HC. In this study, 42% of CD patients were vitamin D deficient 25(OH)D_3_ (≤20 ng/mL), while 29% were insufficient (25(OH)D_3_: 21–29 ng/mL); 12 h after ingesting 50,000 IU of vitamin D_2_, circulating levels of this metabolite were significantly lower in CD compared with HC indicating a significant 30% reduction of the ability to absorb vitamin D_2_. In another study, on 31 CD patients and 15 HC, the capacity of absorbing orally administered vitamin D (5 *μ*g of 25(OH)D_3_/kg body weight) was evaluated; 10% of CD patients showed decreased absorption of 25(OH)D_3_ after 4 and 8 hours [71]. Finally, a wide variability of absorption of vitamin D_2_ was reported in vitamin deficient and insufficient CD patients, but vitamin D_2_ absorption was significantly reduced compared with HC [77].

Several studies evaluated factors influencing vitamin D status hypothesizing reduced sun exposure as cause for hypovitaminosis, since a geographical north-south gradient was noted also for other autoimmune T helper- (Th-) 1-mediated diseases, like multiple sclerosis. The link between this gradient and the pathophysiological mechanisms that involve vitamin D status depends not only on dietary intake but also from UV exposure [83]. Indeed, a negative association between sun exposure and lower levels of 25(OH)D in CD was reported in Indian patients [52] and, most recently, also in Dutch CD [84] where reduced exposure to sunlight (defined as no sunny holidays, no solarium use, and more sun protection) was associated with low 25(OH)D serum levels.

The relationship between sun exposure and the risk of developing CD or UC has been investigated by Nerich et al. [85]. High residential sunlight exposure was associated with a significant decreased risk of CD, but not UC. Four years later, the same group published similar results, that is, an increased incidence of CD with reduced sunlight exposure, in a cohort of women living in France, whereas vitamin D intake was not associated with a risk reduction in CD or UC [86].

Reduced UV exposure seems therefore not only to increase risk for CD, but it also seems associated with a worse outcome of disease. In a recent nationwide North-American study, the influence of UV exposure on hospitalization rates, length of hospital stay, and surgeries was investigated in an impressive number of IBD patients (649,932 CD, 384,267 UC, and 288,894,297 non-IBD controls). Reduced UV exposure led to significantly longer hospitalizations in all groups and to more frequent intestinal surgeries and deaths in CD [87]. Data on 25(OH)D_3_ were not available in this study. The finding that more UV exposure is associated with a minor number of surgical procedures in CD was confirmed in a subsequent study on 481,712 CD-related hospitalizations reporting 67,751 major surgical procedures [88].

Finally, a prospective cohort study of 72,179 women enrolled in the Nurses' Health Study addressed the question if vitamin D hypovitaminosis may, per se, represent a risk factor for the development of IBD. Incident cases of CD and UC were recorded over a follow-up period of 22 years. A 25(OH)D_3_ prediction score based on diet and lifestyle was developed and validated against effectively measured levels of 25(OH)D_3_. The authors showed that higher predicted plasma levels of 25(OH)D_3_ were associated with a significant risk reduction for CD but not for UC, suggesting that vitamin D status may contribute to the pathogenesis of CD [89].

After a series of contradictory and mostly negative studies on vitamin D levels in IBD patients compared with HC, more conclusive data have been produced introducing reference values. However, most of these studies have been aimed to investigate bone and calcium metabolism. Recent large cohort studies investigating UV exposure or vitamin D status estimating the risk to develop IBD have pushed forward our understanding on the potential role of vitamin D in the context of IBD.

## 5. Vitamin D Status and Clinical Outcome in IBD Patients 

Several studies concerning the relationship between vitamin D status and clinical outcome in IBD patients have been published (Table 2). Almost 30 years ago, 25(OH)D_3_ levels in active CD were found to be lower than in quiescent CD [50]. Twenty years later, another study showed that low serum 25(OH)D_3_ levels were predicted by disease duration and activity scores in both, CD and UC [46]. This inverse association between disease activity and serum 25(OH)D_3_ levels was confirmed in a small prospective study in CD [52] and in a retrospective study on a much larger, mixed IBD population [59]. In this latter study, low serum 25(OH)D_3_ levels were associated with higher clinical activity scores in CD and in UC, but not with the risk for medical or surgical hospitalizations. Moreover, regression analysis found that low vitamin D levels were independently associated with quality of life (QoL) in CD patients but not in UC patients. A reduced QoL was reproduced by another study where vitamin insufficient patients had significantly lower QoL scores than those who were sufficient [82]. Finally, in a mixed IBD population, an inverse correlation between serum 25(OH)D_3_ concentrations and fecal calprotectin, a marker for gut inflammation, was found whereas serum CRP as a marker of systemic inflammation did not correlate with 25(OH)D_3_ levels [90].

Conversely, other studies on CD and UC patients failed to show a correlation between serum 25(OH)D_3_ levels and disease activity [60]. The same findings, that is, no association between 25(OH)D_3_ concentrations and disease activity, were published on a pediatric IBD population [54].

Going beyond disease activity, in a prospective study on the largest multicenter cohort involving 3,217 patients, low plasma 25(OH)D_3_ levels (<20 ng/mL) were associated with an increased risk of hospitalizations and surgery for CD as well as for UC patients [81]. In a subset of CD patients, but not UC patients, who normalized vitamin D status, a reduction of CRP levels and the need for hospitalizations was observed.

The likelihood for developing* Clostridium difficile* (Cl) colitis related to vitamin D status was investigated retrospectively. There was an increased risk for developing Cl colitis in patients with low plasma 25(OH)D_3_ levels (<20 ng/mL), and an increase by 1 ng/mL of 25(OH)D_3_ was accompanied by a 4% risk reduction of developing Cl colitis. Lastly, death from Cl colitis occurred in those with lower 25(OH)D_3_ levels compared with survivors [91]. A recent study investigated the relationship between 25(OH)D_3_ concentrations and duration of anti-TNF therapy in IBD patients. Interestingly, low vitamin D levels were associated with loss of response during maintenance therapy in CD patients [92], whereas serum 25(OH)D_3_ levels increased with anti-TNF therapy [93].

The only study that investigated plasma 1,25(OH)_2_D_3_ levels found no association between 1,25(OH)_2_D_3_ levels and CDAI or CAI in Japanese patients [68].

From the above, it appears that low vitamin D is inversely correlated to disease activity documented by clinical scores and surrogate markers of inflammation such as CRP and fecal calprotectin; moreover, low levels were also associated with clinical outcomes, that is, surgery, response to anti-TNF therapy, Cl superinfection, and, finally, death. Inflammation per se has been shown to upregulate conversion from 25(OH)D_3_ to 1,25(OH)_2_D_3_ which may lead to a reduction of available 25(OH)D_3_. In this discussion, an observation of two recent papers may be relevant, coming from orthopaedic surgery, showing an acute reduction of 25(OH)D_3_ levels following a systemic inflammatory response induced by surgery, considering serum 25(OH)D_3_ as a negative acute phase reactant [94, 95].

## 6. Therapeutic Studies* In Vitro* and in Experimental Animals 

As a result of this evidence, vitamin D should be proposed as a therapy for IBD. Several experimental studies, both on animals and IBD patients, have been carried out (Table 3). Starting with the former, in a model of spontaneous colitis, interleukin- (IL-) 10 knock-out (KO) mice on a vitamin D deficient diet showed growth retardation and weight loss, together with a high mortality rate (58% at week 9) compared to mice on a vitamin D sufficient diet; 1,25(OH)_2_D_3_ (0.005 *μ*g/day) supplementation starting from week 2 reduced weight loss and ameliorated histology scores, but vitamin D supplementation after symptom onset at week 7 (1,25(OH)_2_D_3_, 0.2 *μ*g/day) did not induce significant differences compared with untreated animals, except for bowel weight indicating a reduction of inflammation in supplemented animals [96]. In another study, the efficacy of a low calcemic vitamin D analogue (22-ene-25-oxa-vitamin D (ZK156979)) was investigated in 2,4,6-trinitrobenzene sulfonic acid (TNBS) colitis [97]. Treatment was performed with 1,25(OH)_2_D_3_ (0.2 *μ*g/kg) versus ZK156979 (0.1–2.0 *μ*g/kg), both administered intraperitoneally (i.p.) before or after colitis induction. Assessment of inflammation and colitis severity was established by scoring colitis, macroscopic and histological analysis, and measurement of myeloperoxidase activity (MPO) and cytokine levels. The authors found that ZK156978 reduced the severity of TNBS-induced colitis with a potency comparable with that of 1,25(OH)_2_D_3_, downregulating MPO activity, tumor necrosis factor-*α* (TNF-*α*) and interferon-*γ* (IFN-*γ*) tissue levels, and T-box transcription factor (T-bet) expression, together with an increase of interleukin IL-10 and IL-4 tissue concentrations, without calcemic effects.

Laverny et al. [98] studied the effect of an intrarectally administered vitamin D receptor agonist (1*α*,25(OH)_2_-16-ene-20-cyclopropyl-vitamin D_3_; BXL-62) in C57Bl/6 mice with dextran-sodium sulfate- (DSS-) induced (3%) colitis. BXL-62 treatment (1 *μ*g/kg) compared to 1,25(OH)_2_D_3_ (0.3 *μ*g/kg) was superior in preventing weight loss and visible fecal blood, together with better stool consistency and histology scores without inducing hypercalcemia. Another synthetic vitamin D agonist, 1*α*,25(OH)2-19-nor-14,20-bisepi-23-yne-vitamin D_3_ (TX527), has been shown to attenuate inflammation in the DSS model of colitis by downregulating IL-1, IL-6, IFN-*γ*, and TNF-*α* as well as the gastrointestinal glutathione peroxidase 2 [99].

There are three very interesting studies which associate vitamin D or its receptor with intestinal microbiota. First, in Cyp27b1-KO mice, that is, mice unable to produce 1,25(OH)_2_D_3_, an increased susceptibility to DSS colitis was observed [100]. Oral vitamin supplementation reduced weight loss, whereas treatment with antibiotics greatly attenuated colitis. In these mice, a reduced expression of E-cadherin on epithelial and immune cells was observed pointing towards a more “leaky” gut. Moreover, a reduced number of tolerogenic dendritic cells were observed in the gut of Cyp27b1-KO mice. In these mice, as well as in VDR-KO mice, dysbiosis of the microbiota was observed with an increase of the Helicobacteraceae family and a reduction of the Firmicutes and Deferribacteres phyla. The authors concluded that vitamin D (production or its receptor) is involved in the regulation of the gut microbiota. Second, DSS-induced colitis was reduced together with a lower penetration of adherent-invasive* E. coli* (AIEC) in mice on a vitamin-sufficient diet compared to those fed a vitamin D deficient diet. Moreover, vitamin D hypovitaminosis and DSS colitis led to an increase of Bacteroidetes. In the same paper in Caco cells incubated with or without vitamin D and then challenged with AIEC, vitamin D maintained transepithelial resistance and prevented tight junctional protein redistribution [101]. The third paper, that reported changes of the microbiota related to interference in the vitamin D system, assessed susceptibility to DSS colitis in conditional VDR KO mice (deletion restricted to the intestinal epithelial cells), along with Paneth cell quantity and quality by means of quantification of lysozyme and ATG16L1 protein expression. The latter is a protein involved in autophagy, and its genetic variants are well known as risk factors for CD. In this model, an increase of* E. coli* and* Bacteroides,* together with a decrease of butyrate producing bacteria was reported. Supplementing butyrate to IL-10 KO mice reverses reduced VDR and ATG16L1 expression. Similar results, that is, an increased expression of VDR and ATG16L1, were obtained incubating several cell lines with butyrate [21].

Finally, a reduction of intestinal fibrosis, assessed by production of extracellular matrix and total collagen, was seen in mice with TNBS colitis on a vitamin supplemented diet compared to mice fed a vitamin D deficient diet [102]. Moreover, in isolated subepithelial myofibroblasts from the colon, a vitamin D sufficient diet reduced concentrations of TGF-*β*1, Smad 3, p-Smad 3, and collagen I. It was concluded that preventive vitamin D administration reduces fibrosis inhibiting the VDR-mediated TGF-*β*1/Smad 3 pathway.

In the above studies, in various types of spontaneous or chemically induced colitis and in several cell lines, vitamin D and synthetic agonists have been shown to reduce colitis severity and intestinal fibrosis. Vitamin D hypovitaminosis or knocking down Cyp27b1 or VDR had the opposite results. Interestingly, these latter conditions were all associated with changes of the intestinal microbiota.

## 7. Therapeutic Studies in Human* Ex Vivo* Preparations

In an* ex vivo* study on PBMC obtained from IBD patients and incubated in the presence of 1,25(OH)_2_D_3_, a reduction of interferon- (IFN-) *γ* and an increase of IL-10 production were observed in PBMC from UC patients whereas in CD the production of TNF-*α* were reduced [103]. The effect of orally administered vitamin D3 on monocyte-depleted PBMC from vitamin D3-treated (1200 IU vitamin D daily over 1 year) versus placebo-treated patients was investigated [104]. CD4^+^ T-cell proliferation and T-cell cytokine production were assessed. IL-6 production in vitamin D_3_-treated patients increased, whereas TNF-*α*, IFN-*γ*, and IL-4 did not. No change was observed for IL-10 and the percentage of the CD4^+^, CD25^+^, and Foxp3^+^ regulatory T cells compared to placebo. The amount of proliferating CD4^+^ T cells was significantly increased (from 41% to 56%) in the vitamin-D-treated group.

Another* ex vivo* study employed the vitamin D analogue (19-nor-14,20-bisepi-23-yne-1,25(OH)_2_D_3_; TX 527). This analogue significantly inhibited PBMC proliferation and TNF-*α* release in CD and HC [105]. The increase of VDR protein levels after incubation with TX 527 was higher in CD compared with HC. Moreover, in PBMC of both, HC and CD, stimulated with TNF-*α*, a decrease in nuclear NF-*κ*B protein levels together with an increase in cytoplasmic IKB-*α* levels were observed pointing to an inhibition of TNF-*α* induced effects on PBMC exerted by the vitamin D analogue.

The effect of the vitamin receptor agonist BXL-62 on PBMC from CD and UC patients and lamina propria mononuclear cells (LPMC) obtained from biopsies of two CD (ileum) and two UC (colon) patients was investigated [98]. After incubation, in LPS-stimulated PBMC and in activated LPMC from IBD patients, BXL-62 significantly inhibited, with a significantly higher potency compared with 1,25(OH)_2_D_3_, TNF-*α*, IL-6, and IL-12/23p40 transcription and cytokine concentrations measured in culture supernatants without differences between CD and UC.

In PBMC of CD patients, expression of the CYP27B1 gene, that is, the gene that encodes the enzyme that converts 25(OH)D_3_ to 1,25(OH)_2_D_3_, and that of the VDR gene was investigated, showing a higher expression in active compared to inactive disease [93]. Moreover, CD4^+^ T cells incubated in the presence of vitamin D showed a threefold increase of CD25^+^ cells.

Finally, the effect of oral vitamin D supplementation on the maturation and cytokine production of monocyte-derived dendritic cells of CD patients was studied [106]. Compared to placebo-treated CD patients, vitamin D supplementation led to reduced CD80 expression in LPS-stimulated dendritic cells together with reduced production of IL-10, IL-1*β*, and IL-6.

## 8. Therapeutic Studies in Human IBD

There are only few studies with vitamin D addressing the clinical course of IBD (Table 3). In one of these studies, the effect of supplementation of the active form of vitamin D 1,25(OH)_2_D_3_ (aVD, 1000 IU 1.25(OH)_2_D_3_ daily) versus the plain vitamin D 25(OH)D (pVD; 2 × 0.25 *μ*g alphacalcidiol daily) was investigated in CD patients in clinical remission (CDAI < 150) [107]. Both groups received oral calcium supplementation (1000 mg/day). At 6 weeks, the mean CDAI and IBDQ scores, as well as the CRP concentrations, decreased in the aVD-treated group, but not in the pVD-treated group. These differences between the groups however disappeared by week 52. Serum calcium concentrations did not change at any time point. Jørgensen et al. [108] performed a randomized double-blind placebo-controlled multicenter study to assess the benefit of vitamin D3 treatment in CD. They included 94 CD patients in clinical (CDAI < 150) and biochemical remission, randomized to receive 1200 IU of vitamin D3 + 1200 mg of calcium or 1200 mg of calcium alone. During 1-year follow-up, serum 25(OH)D_3_ levels increased significantly in vitamin D-supplemented patients, on average from 27 to 38 ng/mL, but free serum calcium did not change. The relapse rate (defined as increase of CDAI >70 over baseline and CDAI ≥150) was not significantly lowered. Adjustment for the use of azathioprine and smoking resulted in minor changes of the risk estimate. However, the authors concluded that vitamin D might be effective in CD but claimed the need for larger studies.

In an uncontrolled study, 18 active CD patients were initially treated with 1000 IU vitamin D daily over 2 weeks. Thereafter, the dose was escalated (to a maximum of 5000 IU) until a serum concentration of 40 ng/mL of 25(OH)D_3_ was reached [109]. After 24 weeks, a significant reduction of the CDAI and an improvement of the IBDQ score were observed. No differences were observed for CRP, erythrocyte sedimentation rate (ESR), TNF-*α*, IL-17, IL-10, and vascular endothelial growth factor (VEGF). Data on serum calcium levels were not reported.

In this last paragraph, the therapeutic effects of vitamin D supplementation on disease activity mainly given to patients in remission yielded modest results; the daily administered dose ranged in these studies between 1000 and 5000 IU, with an increase of serum vitamin D levels but apparently without hypercalcemia.

## 9. Conclusions

Literature data highlighting the importance of vitamin D in different aspects of immune regulation, for example, in chronic immune-mediated diseases and cancer, suggest considering this metabolite not simply as a vitamin involved in bone and calcium homeostasis but as an autocrine mediator with an active role in numerous physiological processes, particularly in the innate immune system. Since most studies concerning the calcium status in IBD yielded contradictory data, in the most recent literature, the discussion has focused on the possible role of vitamin D as a risk factor for the onset and evolution of gut inflammation. The potential role of 25(OH)_2_D as negative acute phase reactant has yet to be proven in IBD but may explain its frequently reduced levels in active disease. Besides lower vitamin D levels due to reduced UV exposure, genetic induced loss of function of VDR may contribute to defects involving vitamin D pathways. It has been shown in VDR KO animals that this deletion profoundly alters innate immune response and the gut microbiota. Further studies in this field are needed to provide more insight in the link between vitamin D/VDR and bowel inflammation.

Simple vitamin D supplementation does not seem to lead to significant improvement of the clinical course of IBD but may be indicated for a subset of patients. Vitamin D synthetic analogues of vitamin D seem to be more promising, at least in animal studies and in* ex vivo* experiments.

## Figures and Tables

**Table 1:** Genetic polymorphisms and IBD (chronological order).

Author	Year	Population	Investigated gene polymorphisms	Main findings
Single- or multicenter studies				

Simmons et al. [23]	2000	England 158 UC, 245 CD, 164 CRADC	VDR: TaqI, ApaI, FokI	TaqI polymorphism (“tt” genotype) more frequent in CD compared to UC or controls

Martin et al. [24]	2002	Germany, 95 CD, 93 UC, 119 HC	VDR: TaqI	TaqI (“tt” genotype) significantly more frequent in fistulizing and stenosing CD

Dresner-Pollak et al. [36]	2004	Israel, 228 CD (129 Ashkenazi and 99 non-Ashkenazi), 151 UC (72 Ashkenazi, 79 non-Ashkenazi), 495 HC (352 non-Ashkenazi and 143 Ashkenazi)	VDR: BsmI	BB genotype more frequent in Ashkenazi UC compared to Ashkenazi HC

Noble et al. [34]	2008	United Kingdom, 286 CD, 154 UC, 240 HC	VDR: TaqI, ApaI	Overall no differences between CD, UC, and HC for TaqI and ApaI. TaqI variants more frequent in male IBD patients compared to (male) HC

Naderi et al. [37]	2008	Iran, 150 UC, 80 CD, 150 HC	VDR: ApaI, TaqI, BsmI, FokI	FokI polymorphism significantly higher in UC and CD. Frequency of polymorphic “f” allele and f/f genotype higher in UC and CD comparing with HC

Pluskiewicz et al. [30]	2009	Poland, 47 UC, 47 HC	VDR: TaqI, BsmI, ApaI	No differences between UC and HC

Hughes et al. [29]	2011	Ireland, 660 IBD, 699 HC	VDR: ApaI, TaqI, BsmI, FokI	Borderline significance for heterozygous carriage of the FokI allele

Pei et al. [31]	2011	China, 218 UC, 251 HC	VDR: ApaI, TaqI, BsmI, FokI	Only Bb genotype of the BsmI variant associated with UC; frequency of the BsmI polymorphic allele (B) increased in UC

Eloranta et al. [42]	2011	Switzerland, 404 CD, 232 UC, 248 HC	DBP: rs 7041, rs 4588	Significantly reduced frequency of the 420 variant Lys in IBD compared to controls

Bentley et al. [35]	2011	New Zealand, 449 CD, 448 UC, 482 HC	VDR: FokI, TaqI	No overall differences, only a higher minor allele frequency for TaqI, in male CD and UC compared to HC

Luo et al. [33]^*^	2013	China, 19 CD, 122 HC	VDR: ApaI, TaqI, BsmI	No significant differences in the frequencies of TaqI, BsmI, and ApaI polymorphisms

Xia et al. [32]	2014	China 382 UC, 489 HC	VDR: ApaI, TaqI, BsmI, FokI	No difference between UC and HC. The mutant allele C and genotype TC + CC of FokI were significantly increased in patients with mild and moderate UC compared to severe UC. The frequency of AAC haplotype was statistically lower in UC than HC (AAC haplotype formed by the VDR BsmI, ApaI, and TaqI gene might engender a reduced risk of UC attack)

Meta-analyses				

Xue et al. [38]	2013	ApaI: 1024 CD, 974 UC, 1551 HC FokI: 1187 CD, 1221 UC, 1746 HC BsmI: 721 CD, 813 UC, 1642 HC TaqI: 1568 CD, 1515 UC, 2152 HC	VDR: ApaI, TaqI, BsmI, FokI	FokI “ff” genotype associated with a significant risk for UC in Asians; TaqI “tt” genotype associated with an increased risk for CD in Europeans and with an increased risk for CD and UC in Asian males. ApaI “a” allele confers protection from CD

Wang et al. [39]	2014	ApaI: 940 CD, 962 UC, 1468 HC FokI: 1098 CD, 1217 UC, 1676 HC BsmI: 713 CD, 799 UC, 1616 HC TaqI: 1553 CD, 1500 UC, 2145 HC	VDR: ApaI, TaqI, BsmI, FokI	ApaI, BsmI, and FokI are not significantly associated with IBD. Significant association between TaqI polymorphism and IBD risk. In subgroups, ApaI increases the overall CD risk and BsmI increases this CD risk only in East Asians, whereas TaqI reduces the risk for UC especially in Caucasians

CD: Crohn's disease; UC: ulcerative colitis; CRADC: cadaveric renal allograft donor controls; PCR: polymerase chain reaction; IBD: inflammatory bowel disease; HC: healthy controls; DBP: vitamin-D-binding protein; VDR: vitamin D receptor.

^*^Article in Chinese.

**Table 2:** Vitamin D versus disease activity and outcome in IBD (chronological order).

Author	Year	Population	Methodology	Main findings
Harries et al. [50]	1985	U.S.A 40 CD 20 UC 9 HC	Single-center cohort; CD divided into 2 groups (undernourished and well nourished); 2 control groups: 20 well-nourished UC and 9 HC	25(OH)D_3_ significantly lower in CD with active disease versus inactive disease (*P* < 0.05)

Tajika et al. [46]	2004	Japan 33 CD, 11 UC, 15 HC	Single-center cohort; 25(OH)D_3_ and disease activity assessed by CDAI and IOIBD score	Serum 25(OH)D_3_ significantly related to disease duration (*r* = 0.46, *P* = 0.003), CDAI (*r* = 0.44, *P* = 0.005), IOIBD score (*r* = 0.30, *P* < 0.05), serum ferritin (*r* = 0.34, *P* = 0.03), CRP (*r* = 0.34, *P* = 0.03)

Joseph et al. [52]	2009	India 34 CD, 34 HC	Single-center cohort; disease activity evaluated by HBI in CD	Serum 25(OH)D_3_ in CD significantly lower versus controls (*P* < 0.05). Disease activity correlated negatively with 25(OH)D_3_ level (*P* < 0.004). 25(OH)D_3_ levels were comparable to controls in mild CD but were significantly lower in moderate and severe CD

Nakajima et al. [68]	2011	Japan 47 CD, 40 UC, 41 HC	Single-center cohort; disease activity measured using CAI/CDAI scores	No decrease 1,25(OH)_2_D_3_ in CD with high CDAI No significant correlation between serum 1,25(OH)_2_D_3_ levels and CAI or CDAI in UC or CD

Ulitsky et al. [59]	2011	U.S.A. 504 IBD (403 CD, 101 UC)	Single-center cohort; retrospective observational study HRQOL measured with SIBDQ, disease activity measured using HBI/UCDI scores	25(OH)D_3_ deficiency significantly associated with lower SIBDQ (*P* = 0.002) and higher mean HBI/UCDI (*P* = 0.002) in IBD versus vit D sufficient patients. Analyzed separately, vit D deficiency associated with lower HRQOL scores only in CD (*P* = 0.04), not in UC

El-Matary et al. [54]	2011	Canada 60 IBD (39 CD, 21 UC)	Cross-sectional pediatric study. Disease activity measured by PCDAI e PUCAI	No correlation between PCDAI and serum 25(OH)D_3_. Marginal evidence against the null hypothesis (*P* = 0.05) between serum 25(OH)D_3_ and PUCAI, but without statistical significance

Hassan et al. [60]	2013	Iran 60 IBD (34 UC, 26 CD)	Cross-sectional study. Disease activity measured by CDAI and Truelove index	Serum vit D lower in active versus inactive disease (non significantly). VitD deficiency was not associated with IBD activity (also considering CD and UC separately), however was associated with a history of IBD related intestinal surgery

Ananthakrishnan et al. [81]	2013	U.S.A. 3,217 IBD (55% CD, 45% UC)	Multicenter cohort; 25(OH)D_3_: Normal (>30 ng/mL), Insufficient (20–29.9 ng/mL) or Deficient (<20 ng/mL)	IBD-related surgery: CD: 10% patients never vitamin D deficient versus 13% vitamin D insufficient versus 17% vitamin D deficient. UC: vitamin D deficiency associated with elevated risk of surgery and hospitalization with effect similar to CD; no statistical significance in patients vitamin D insufficient. Normalization of 25(OH)D_3 _associated with reduction in the risk of related surgery but not in UC

Zator et al. [92]	2014	U.S.A. 101 IBD (74 CD, 27 UC)	Retrospective single-center cohort; patients on anti-TNF therapy evaluated for loss of response; 25(OH)D_3_ insufficiency: <30 ng/mL	Patients with insufficient vitamin D demonstrated earlier cessation of anti-TNF-*α* therapy (*P* = 0.04). This effect was significant in patients who stopped treatment for loss of response, stronger for CD than UC (*P* = NS)

Ananthakrishnan et al. [91]	2014	U.S.A. 3188 IBD patients (45% UC, 55% CD)	Retrospective multi-center analysis of 25(OH)D_3_ in 35 patients who developed CDI	25(OH)D_3_ level was significantly lower in IBD who developed CDI compared to non-CDI-IBD (*P* = 0.002). Levels below 20 ng/mL were associated with a two-fold increase in risk of CDI. 25(OH)D_3_ level was an independent predictor of CDI

Ham et al. [93]	2014	U.S.A. 37 CD	Prospectively collected samples for 25(OH)D_3_ analysis; assessment of HBI and CRP PBMC tested for VDR, Cyp	25(OH)D_3_ levels lower in patients with active disease versus inactive disease, 25(OH)D_3_ correlated with HBI (not with CRP) PBMC: mean gene expression of VDR and CypB1 higher in active disease

Garg et al. [90]	2013	Australia 40 CD 31 UC 23 HC	Assessment of 25(OH)D_3_, fecal calprotectin and CRP	Inverse correlation between serum 25(OH)D_3_ and fecal calprotectin in CD and UC patients, but not with CRP

Hlavaty et al. [82]	2014	Slovakia 141 CD 49 UC	SIBDQ assessment in vitamin D sufficient or -deficient patients and in vitamin supplement (800 IU/day for 3 months) patients	SIBDQ was significantly better in vitamin D-sufficient patients; vitamin D supplements did not influence vitamin D status or sIBDQ

Govani et al. [88]	2015	U.S.A. 67,751 CD	Retrospective, national, analysis of UV exposure and inpatient surgery risk	UV exposure protective for inpatients surgery

Abbreviations: CD: Crohn's disease; UC: ulcerative colitis; HC: healthy controls; IBS: irritable bowel syndrome; IBD: inflammatory bowel disease; CDAI: Crohn's Disease Activity Index; IOIBD: international organization for the study of inflammatory bowel disease score; CAI: Lichtiger's clinical activity index; 25(OH)D_3_: 25-Hydroxycholecalciferol; 1,25(OH)_2_D_3_: 1,25dihydroxycholecalciferol; SIBDQ: Short IBD Questionnaire; HBI: Harvey-Bradshaw index; UCDI: Ulcerative colitis disease activity index; HRQOL: health-related quality of life; PCDAI: pediatric Crohn's disease activity index; PUCAI: pediatric ulcerative colitis activity index; CDI: Clostridium difficile infection; CRP: C-reactive protein; UV: ultraviolet; TNF: tumor necrosis factor; PBMC: peripheral blood mononuclear cells; Cyp: Cyp27b1 gene; VDR: vitamin D receptor.

**Table 3:** Therapeutic studies in experimental and human IBD (chronological order).

Author	Year	Species/cells	Investigational agent	Methodology	Main findings
Animal and *in vitro* studies					

Cantorna et al. [96]	2000	IL-10 KO mice	1,25(OH)_2_D_3_ p.o.	Exp. 1. Vit. D-deficient IL-10 KO mice versus vit. D-sufficient mice (treated with cholecalciferol); Exp. 2. Vit. D-deficient IL10 KO mice versus 1,25(OH)_2_D_3_- treated; Exp. 3. Vit. D treatment after onset of GI symptoms	Vitamin D sufficiency prevents enterocolitis in IL-10 KO mice up to 13 weeks; 1,25(OH)_2_D_3_ treatment ameliorates inflammation

Daniel et al. [97]	2006	BALB/c mice	TNBS colitis; 22-ene-25-oxa-vitamin D (ZK156979) i.p. (vitamin D analogue)	Treatment with ZK156979 versus 1,25(OH)_2_D_3_ before or after induction of colitis with TNBS; investigation of tissue MPO, TNF-*α*, IFN-*γ*, T-bet, IL-10, and IL-4	ZK156979 versus 1,25(OH)_2_D_3_ prevents or ameliorates TNBS colitis decreasing pro-inflammatory and increasing anti-inflammatory cytokines

Laverny et al. [98]	2010	C57BL/6 mice	DSS-colitis, 1*α*,25(OH)_2_-16-ene-20-cyclopropyl-vitamin D3 (BXL-62) (=VDR agonist) intrarectally	Daily administration of BXL-62 versus 1,25(OH)_2_D_3_; Macro- and microscopic scoring; mucosal concentrations of TNF-*α*, IL-12/23p40, IL-6, and IFN-*γ* and assessment of mRNA	Higher potency of BXL-62 versus 1,25(OH)_2_D_3_ in reducing tissue inflammation

Verlinden et al. [99]	2013	C57BL/6 mice	DSS- colitis 1*α*,25(OH)_2_-19-nor-14,20-bisepi-23-yne-vitamin D3 (TX527)	Histological examination; measurement of transcript levels of cytokines (IL-1, IL-6, IFN-*γ*, and TNF-*α*)	TX527 reduced “clinical” disease scores and attenuated histological scores, downregulation of transcript levels of inflammatory cytokines

Ooi et al. [100]	2013	C57BL/6 mice Cyp KO VDR KO	1,25(OH)_2_D_3_ p.o.	DSS colitis; characterization of gut microbiota, and gut macrophages; E-cadherin expression	Lower expression of E cadherin and tolerogenic macrophages Less beneficial microbiota in KO mice Vitamin D treatment ameliorates colitis and reduces Helicobacteraceae

Wu et al. [21]	2014	Conditional VDR KO and IL-10 KO mice DSS-colitis cells: MEF, SKCO15, HCT116 human tissue	DSS colitis BUT feeding in IL-10 KO	VDR KO: colitis evaluation, pyrosequencing for microbiota, Paneth cells, lysozyme production, autophagy MEF (VDR^−/−^ VDR^+/−^ VDR^+/+^) and VDR knockdown in SKCO15 with evaluation of ATG16L1 and LC3B proteins IL-10 KO: VDR and ATG16L1 expression with or w/o BUT feeding Human tissue (UC, inflamed versus normal) VDR, ATG16L1, Bacteroides concentration (FISH) HCT116 and HIEC: VDR expression with and w/o incubation with BUT	Conditional VDR KO mice: worse colitis, increased *E. coli* and Bacteroides (*B. fragilis*), and decreased BUT-producing bacteria; less and abnormal Paneth cells and reduced lysozyme and ATG16L1 protein; in SKCO15 and MEF reduced expression of ATG16L1 and LC3B proteins In UC: reduced expression of VDR and ATG16L1, increase of Bacteroides; BUT increases VDR expression in HIEC and HCT116

Tao et al. [102]	2014	C57BL/6 mice	TNBS-colitis Vitamin D sufficient or deficient diet	At week 14, assessment of ECM and total collagen production, together with determination in isolated colonic SEMF, of expression of VDR, *α*-SMA, and Collagen I in normal SEMF	Histological scoring, ECM, and collagen production in the colon reduced in vitamin D supplemented mice; in SEMF decreased levels of TGF-*β*1, Smad-3, p-Smad3, and Collagen I and induced VDR expression and decreased TGF-*β*1-induced *α*-SMA and Collagen I expression

Assa et al. [101]	2015	Caco cells C57BL/6 mice	DSS- colitis Vitamin D sufficient or deficient diet 1,25(OH)_2_D_3_ for Caco	Caco cells incubated with or w/o 1,25(OH)_2_D_3_ challenged with AIEC C57BL/6 mice on normal or low 1,25(OH)_2_D_3_ diet infected with AIEC	1,25(OH)_2_D_3_ protects Caco cells against AIEC induced loss of TER and TJ protein redistribution 1,25(OH)_2_D_3_ reduces DSS colitis and AIEC invasion low vitamin D diet and DSS colitis increased Bacteroides

*In vivo* and *ex vivo* studies in IBD patients					

Stio et al. [105]	2007	4 CD and 4 HC	TX 527 [19-nor-14,20-bisepi-23-yne-1,25(OH)_2_D_3_], Vitamin D analogue	Single-center, *ex vivo* study; experimental study on PBMC of CD patients	TX 527 inhibits TNF-*α* mediated effects on PBMC and the activation of NF-*κ*B; its action is mediated by VDR

Miheller et al. [107]	2009	37 CD	Group A treated with aVD versus group B treated with pVD	Single-center study; evaluation of bone parameters and CDAI, CRP, and SIBDQ after 6, 12, 52 weeks	In aVD, after 6 weeks (but not at 52 weeks) a significant reduction of CDAI, IBDQ, and CRP together with a significant change of bone parameters

Ardizzone et al. [103]	2009	9 UC, 8 CD	1,25(OH)_2_D_3_	Single-center *ex vivo* study; PBMC with or without calcitriol; determination of TNF-*α*, IFN-*γ*, IL-2, and IL-10	In UC PBMC 1,25(OH)_2_D_3_ reduced IFN-**γ** and enhanced IL-10 production In CD PBMC 1,25(OH)_2_D_3_ reduced TNF-*α* production

Jørgensen et al. [108]	2010	94 CD	Vitamin D3 versus placebo	Multi-center randomized double-blind placebo-controlled study; 1200 IU vit D3/day or placebo; estimation of clinical relapse rate	Vit. D3 significantly increased serum vit. D levels, but the decrease of relapse was not significant (13% versus 29%, *P* = 0.06)

Bendix-Struve et al. [104]	2010	108 CD	Vitamin D3 versus placebo	Randomized, placebo-controlled, clinical trial After 0, 36, and 52 weeks, PBMC tested in 10 patients treated with Vitamin D3 (1200 IU/day) and in 10 patients treated with placebo for cytokine production and proliferation	Vit. D3 treatment of CD patients increased the IL-6 levels and enhance the CD4^+^ T-cell proliferation

Laverny et al. [98]	2010	22 CD, 21 UC	1*α*,25(OH)_2_-16-ene-20-cyclopropyl-vitamin D3 (BXL-62)	*Ex vivo* preparations of PBMC (+LPS) and (CD2/CD28 activated)-LPMCs incubated with or without BXL-62. Determination of mRNA and protein concentrations of TNF-*α*, IL-12/23p40, IL-6, and IFN-*γ*	Higher anti-inflammatory potency compared to 1,25(OH)_2_D_3_ demonstrated by the significantly more potent inhibition in PBMC and in LPMCs of the proinflammatory cytokines TNF-*α*, IL-12/23p40, IL-6, and IFN-*γ*

Yang et al. [109]	2013	18 CD	Vitamin D3	Open-label prospective clinical trial over 24 weeks, multi-center study; vitamin D3 at 1000 IU/day; dose increase every two week of 1000 IU/day up to 5000 IU/day to achieve serum 25(OH)D_3_ >40 ng/mL	Vit. D3 supplementation significantly raised serum 25(OH)D_3_, reduced CDAI scores, and improved IBDQ scores

Bartels et al. [106]	2014	10 CD	Vitamin D3	Single-center study, oral vitamin D supplementation (or placebo) and assessment of maturation marker expression and cytokine production of monocyte-derived dendritic cells	Dendritic cells from vitamin supplemented CD patients exhibited reduced expression of CD80 and reduced production of the cytokines IL-10, IL-1*β*, and IL-6

Ham et al. [93]	2014	PBMC	Incubation of CD4^+^ with vit D 50 nM	Determination of CD25^+^ and CD39^+^ cells	3-fold increase of CD25^+^ cells, CD39^+^ unchanged

CD: Crohn's disease; UC: ulcerative colitis; HC: healthy controls; vit: vitamin; p.o.: per os; GI: gastrointestinal; KO: knock-out; TNBS: 2,4,6-trinitrobenzene sulfonic acid; i.p.: intraperitoneal; DSS: dextran sodium sulfate; 25(OH)D: 25-hydroxycholecalciferol; 1,25(OH)_2_D_3_: 1,25-dihydroxycholecalciferol; vitamin D3 (vit D3): cholecalciferol; VDR: vitamin D receptor; MEF: mouse embryonic fibroblasts; AIEC: adherent-invasive *Escherichia coli*; TER: transepithelial electrical resistance; TJ: tight-junction; aVD: active vitamin D (1,25(OH)_2_D_3_); pVD: plain vitamin D (25(OH)vitamin D); CDAI: Crohn's disease activity index; CRP: C-reactive protein; SIBDQ: Short IBD questionnaire; PBMC: peripheral blood mononuclear cells; LPS, lipopolysaccharide; LPMCs: lamina propria mononuclear cells; IBDQ: IBD questionnaire; IL: interleukin; Cyp: Cyp27b1 gene; IFN: interferon; TNF: tumor necrosis factor; BUT butyrate; SEMF: subepithelial myofibroblasts; ECM: extracellular matrix; *α*-SMA: alpha smooth muscle actin; FISH: fluorescent in situ hybridization; HIEC: human intestinal epithelial cells; ATG16L1: autophagy related 16-like 1 (*S. cerevisiae*); LC3B: autophagy-related protein LC3B; SKCO15: human colorectal adenocarcinoma cells; HCT116: human colon cancer cell.
